# Minimally Invasive Surgery in Chronic Subdural Hematoma: Prognosis and Recurrence Factors of 516 Cases in a Single Center

**DOI:** 10.3390/jcm11051321

**Published:** 2022-02-28

**Authors:** Min Xu, Weiguo Tan, Wenhua Wang, Dongdong Wang, Wei Zeng, Cunzu Wang

**Affiliations:** 1 Department of Neurosurgery, Kunshan Hospital of Traditional Chinese Medicine (Kunshan Affiliated Hospital of Nanjing University of Chinese Medicine), Kunshan 215300, China; fsyy00870@njucm.edu.cn (M.X.); titantan_doc@163.com (W.T.); wwhpb176@sina.com (W.W.); 2 Department of Neurosurgery, Northern Jiangsu People’s Hospital, Yangzhou 225001, China; aswangdadong@163.com (D.W.); zwneurosurgery@sina.cn (W.Z.)

**Keywords:** chronic subdural hematoma, YL-1 puncture needle, risk factor, prognosis

## Abstract

Objective: To investigate the effects of minimally invasive surgery (MIS) using a novel YL-1 puncture needle and summarize the risk factors of recurrence in chronic subdural hematoma (CSDH). Methods: We performed a retrospective analysis in 516 hospitalized patients with CSDH from January 2013 to December 2018 in Northern Jiangsu People’s Hospital. Patients’ gender, age, history of trauma, use of anticoagulants, history of disturbed liver or renal function, history of heart disease, history of malignant tumor, history of diabetes, hemodialysis, coagulopathy, alcoholism, imaging indicators, and postoperative application of urokinase or atorvastatin were recorded. Recurrence is defined by imaging examination with or without clinical presentation three months after discharge. Results: In total, 483 patients (93.60%) benefited from MIS by YL-1 needle. Gender, age, history of head trauma, history of disturbed liver function, history of heart disease, history of malignant tumor, history of diabetes, history of hemodialysis, coagulopathy, alcoholism, hematoma location, hematoma densities, septum formation, maximum thickness, encephalatrophy, and use of atorvastatin and urokinase were shown to be non-significantly associated with postoperative recurrence (*p* > 0.05). The use of anticoagulants was significantly associated with postoperative recurrence (*p >* 0. 05). Logistic analysis showed that the use of anticoagulants is an independent factor predicting postoperative recurrence (*p >* 0. 05). Conclusions: The novel YL-1 puncture needle turned out to be a safe and effective minimally invasive surgery, and the use of anticoagulants is an independent risk factor predicting postoperative recurrence in CSDH, which can provide MIS and early therapeutic strategies for neurosurgeons.

## 1. Introduction

Chronic subdural hematoma (CSDH) is a common clinical disease treated by neurosurgeons. The incidence is about 14.1 of 100,000 per year in the general population [1,2]. Males represent approximately 75% of the cases, and two-thirds have a clear history of trauma [3]. In developing countries, the incidence reaches 0.0074% in the elderly group, accounting for nearly 10% of intracranial hemorrhage [4]. Moreover, elderly patients have a 10-fold increase in the risk of developing CSDH depending on their anticoagulation status [5]. Because of the increasing rise towards life expectancy and population aging, higher incidence and diagnostic innovation bring new challenges to our treatments.

At present, there are different kinds of treatment techniques, including conservative treatment, twist drill or burr-hole surgery, and craniectomy [6,7]. Despite technological advances in the neurosurgical field, there has been little improvement regarding the surgical technique for CSDH, and controversy still remains regarding the concrete therapeutics and mechanisms in CSDH. However, surgical treatments are still the main solutions for progressive hematomas [8]. Although the commonly used techniques are relatively safe and simple, the recurrence rate still ranges from 3% to 35% [9]. Factors that influence the recurrence of CSDH include older age, poor neurological status, cerebral atrophy, large hematoma, chronic alcohol use, use of anticoagulants, disturbed liver or renal function, meningeal dissemination of malignant tumor, and surgical technique used [10,11].

In order to gain an MIS treatment as well as good recovery, early intervention and minimally invasive surgery are often recommended for elderly patients by neurosurgeons. A modified technique named YL-1 puncture needle has been covered often in the literature [11,12,13]. The technique of the YL-1 puncture needle has been introduced and widely used in the center of Northern Jiangsu People’s Hospital. In this research, we performed a retrospective analysis of 516 cases to evaluate the effects of this modified YL-1 technique and summarize the risk factors of recurrence.

## 2. Patients and Methods

### 2.1. Patients Collection

**Figure 1:**
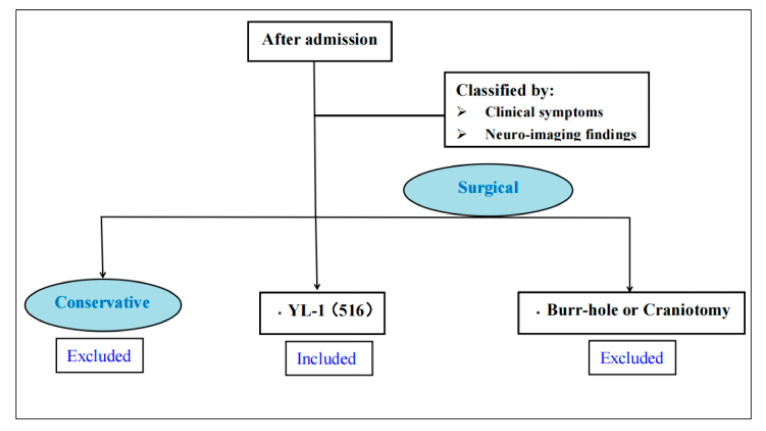
Flow diagram of study selection.

In this research, we collected 516 CSDH patients from January 2013 to December 2018 in Northern Jiangsu People’s Hospital. This study has been approved by Institutional Review Board (IRB) of Northern Jiangsu People’s Hospital, and researches have been declared to follow the principles of the Declaration of Helsinki. The inclusion criteria were defined as follows: all the CSDH patients were treated in surgery of YL-1 needle. Conservative treatments and craniectomy were excluded (Figure 1). Patients’ gender, history of trauma, age, use of anticoagulants, history of disturbed liver or renal function, history of heart disease, malignant tumor, history of diabetes, hemodialysis, coagulopathy, alcoholism, imaging indicators, postoperative application of urokinase or atorvastatin were recorded. Recurrence is defined by imaging examination with or without clinical presentation three months after discharge.

### 2.2. Hospitalized Flow and Surgical Procedure

Based on clinical symptoms and neuro-imaging findings, patients after admission were brought into hospitalized flowchart. Most patients had strong chief complaints (such as symptoms of limb fatigue or headache) or obvious mass effects (such as compression of brain tissues or displacement of midline structure) on Computer Tomography (CT) scans. Surgical treatments were taken into primary consideration. Among surgeries, minimally invasive surgery of YL-1 puncture needle was selected for CSDHs in this center.

**Figure 2:**
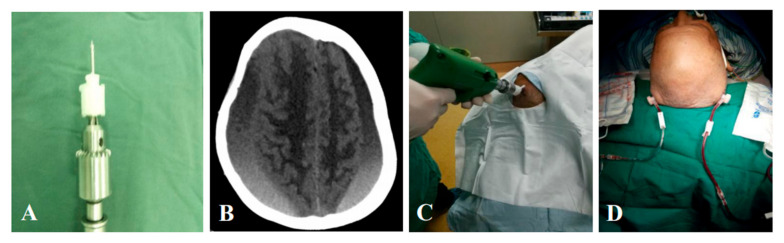
Operation procedure of YL-1 puncture needle. (**A**) Fixation of the YL-1 puncture needle in an electric trepanning drill. (**B**) Preoperative CT scanning. (**C**) Drilling into the hematoma cavity. (**D**) Postoperative drainage.

The YL-1 needle (2 cm) was chosen in treating CSDHs. Based on head CT or Magnetic Resonance Imaging (MRI), the puncture site was selected on a thicker location of hematoma, but avoiding arteria meningea media. After routine disinfection and local anesthesia, the puncture needle (2 cm) was connected to an electric drill (Figure 2A), then a vertical puncture broke through the dura mater (Figure 2C), connected to external tube, and then hematoma fluid flowed out. Either 250 or 500 mL normal saline was conventionally used to wash hematoma cavity (Figure 2D) until the drainage fluid became clear. CT scanning should be performed to check the residual hematoma one day after operation. In addition, urokinase (30,000–50,000 IU) might be given to help dissolute hematoma, and even reduce the rate of recurrence. After 2 to 4 days’ drainage, the removal of YL-1 needle could be evaluated by drainage situation or CT scanning [14].

### 2.3. Statistical Analysis

The clinical indicators listed were analyzed by SPSS17.0 software (SPSS Inc., Chicago, IL, USA). The variables between groups were compared using methods including the *t*-test and the chi-squared (*χ*^2^) test. Then, the statistically significant factors from the univariate factor analysis were screened into the logistic regression model and further analyzed recurrence factors of CSDH. Statistical analysis was conducted on the 95% confidence level. A *p*-value less than 0.05 was considered statistically significant.

## 3. Results

### 3.1. Patients

The 516 CSDH patients were 67.09 ± 11.77 (14–98) years old; 429 were male and 87 female, and 24 had bilateral hematomas. In the statistics of past history, 381 patients (73.80%) had a history of trauma, 22 patients (4.26%) had once used anticoagulants, 11 patients (2.13%) had a history of disturbed liver function, 48 patients (9.30%) had a history of heart disease, 22 patients (4.26%) had malignant tumors, 45 patients (8.72%) had a history of diabetes, 6 patients (1.16%) had a history of hemodialysis, 25 patients (4.84%) had a history of coagulopathy, and 1 patient (0.19%) had a history of alcoholism. In imaging indicators, 55 patients (10.66%) had encephalatrophy, 135 patients (26.16%) had bilateral hematomas, while 381 patients (73.84%) had unilateral hematoma, 265 patients (51.36%) had mixed densities, and 16 patients (3.10%) had septum formation in the hematoma based on CT scanning or MR imaging. The mean maximum thickness of hematoma was 21.59 ± 6.34 mm (5–45 mm). All patients accepted surgery using the YL-1 puncture needle (610 times). Of the 135 patients who had bilateral hematomas, 94 patients accepted bilateral surgeries and 41 patients accepted unilateral surgery. Atorvastatin was taken by 360 patients after the operation and urokinase was used by 462 patients. In total, 33 patients (6.40%) recurred after leaving hospital.

### 3.2. Recurrence Factors of CSDH

**Figure 3:**
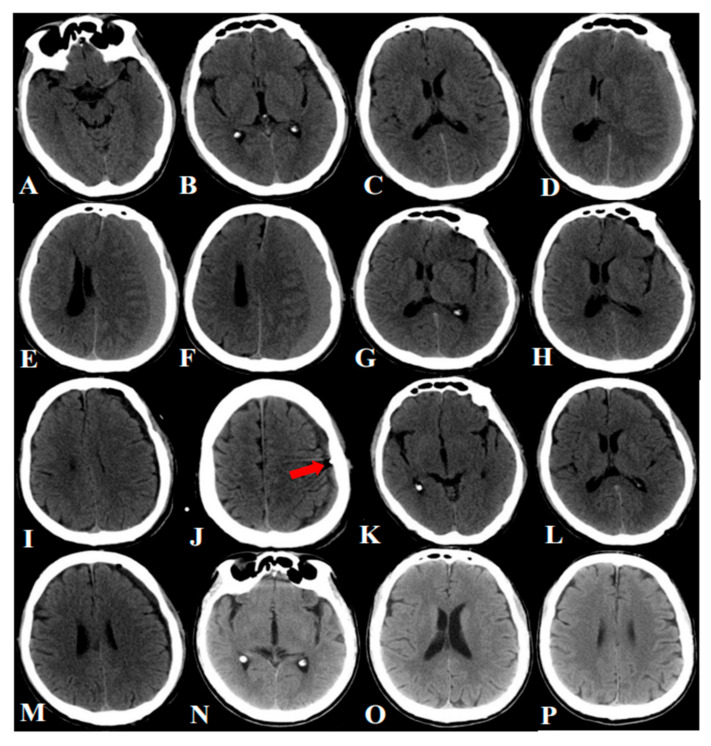
CT imagines of YL-1 puncture needle. (**A**–**C**) CT scanning after head trauma (three months before hospitalization). (**D**–**F**) CT scanning after admission. (**G**–**J**) CT scans one day before operation. (**K**–**M**) CT scanning at discharge. (**N**–**P**) Follow-up CT scanning (3 months).

All patients were treated in minimally invasive surgery using the YL-1 puncture needle (Figure 3). They were classified as divided into two groups: recurrence group (n = 33) and no recurrence group (n = 483). In total, 483 patients (93.60%) benefited from minimally invasive surgery using the YL-1 puncture needle. Gender (*p* = 0.218), age (*p* = 0.482), history of head trauma (*p* = 0.881), history of disturbed liver function (*p* = 0.520), history of heart disease (*p* = 0.534), history of malignant tumor (*p* = 0.644), history of diabetes (*p* = 0.758), history of hemodialysis (*p* = 0.614), history of coagulopathy (*p* = 0.372), history of alcoholism (*p* = 0.794), hematoma location (*p* = 0.795), hematoma densities (*p* = 0.511), septum formation (*p* = 0.615), maximum thickness (*p* = 0.960), encephalatrophy (*p* = 0.380), and use of atorvastatin and urokinase (*p* = 0.504, *p* = 0.836) were shown to be non-significantly associated with postoperative recurrence. The use of anticoagulants was significantly associated with postoperative recurrence (*p* < 0.05) (Table 1).

### 3.3. Logistic Analysis of Recurrence Factors

After univariate analysis, the use of anticoagulants was significantly associated with postoperative recurrence. Then, the statistically significant factor was screened into the logistic regression model and recurrence factors of CSDH (Table 2) were analyzed further. Logistic analysis showed that the use of anticoagulants is an independent factor predicting postoperative recurrence (*p* < 0.05) (Table 2). 

## 4. Discussion

Chronic subdural hematoma is one type of hematoma common among the elderly. Males represent approximately 75% of the cases, and two-thirds have a clear history of trauma [3]. In recent years, the mechanisms of the formation of CSDH have been extensively researched but have still not been identified completely. The introduction of modern imaging methods and operative methods help improve the management of CSDHs. However, the treatment of CSDHs continues to be a challenge. Series of studies have suggested that CSDH is caused based on the theory of dysfunctional angiogenesis and inflammation [15,16]. Angiogenesis was suppressed by blood leakage of the neomembrane and local inflammation. In addition, local inflammation prevented leaked blood from being absorbed. This is the main cause of repeated hemorrhages into the subdural space and expansion of the hematoma [17,18].

Due to the mechanism above, drug treatment (such as atorvastatin) has been reported most often in the literature. Drug treatments were introduced by several centers and have been proved to be safe and effective methods on CSDH [19]. Due to the progressive hematomas of CSDH, surgical treatments are still the main solutions for those with symptoms of increased ICP or brain compression. In order to achieve an MIS treatment and good clinical recovery, multiple modified methods were applied in practice work, in which the novel YL-1 hollow needle aspiration drainage system had been proved to be not obviously different from burr-hole craniotomy in the curative effects of CSDH [2,11]. The technique of the YL-1 puncture needle has been introduced and widely used in the center of the Northern Jiangsu People’s Hospital. In this research, we performed a retrospective analysis to evaluate the effects of this modified YL-1 technique and summarize the risk factors of recurrence.

In this retrospective study, 93.60% of patients (483) benefited from the MIS method, which is consistent with that (84.5, 89.69, 94.4%) in the literature [8,14]. Male sex and age were still controversial independent risk factors of CSDH recurrence. The authors showed that men probably had more trauma and more frequent complications than women. In addition, brain atrophy of older patients may contribute to the development of CSDH [20]. In the present study, gender, age, and history of head trauma were shown to be non-significantly associated with postoperative recurrence, as well as in the past history, such as a history of disturbed liver function, heart disease, malignant tumor, diabetes, hemodialysis, coagulopathy, and alcoholism (*p* > 0.05). Several groups have already described hematoma volume or thickness as predictors of CSDH recurrence. Less residual hematoma volume may result in the chance of an early cure. In addition, atorvastatin has been proved to be safe and effective in conservative patients [2,19]. However, it is still controversial as to whether oral atorvastatin is of benefit to the prevention of recurrence after surgery. Meanwhile, glucocorticoid, especially dexamethasone, has been used to manage CSDH. Recent meta-analysis after the DEX-CSDH trial had shown some benefits in terms of recurrence but no benefit in terms of mortality with significantly higher adverse events [21].

In the current study, we found that anticoagulant agents such as warfarin increased CSDH recurrence. This finding is a matter of controversy in the literature as some studies found that the administration of anticoagulants is not predictive of recurrence [22,23,24], while others found an active effect of antiplatelet or anticoagulant therapy on the recurrence rate [25,26]. Our finding was in line with the results of the latter groups. It is considered that antiplatelet and anticoagulant agents prevent the formation of clots. This mechanism may contribute to microhemorrhage into the hematoma cavity and be presumably involved in CSDH recurrence. After univariate analysis, the use of anticoagulants was significantly associated with postoperative recurrence, and the multivariate analysis found anticoagulants to be an independent risk factor.

In addition, this study has several limitations. The main limitation is the retrospective study design and the reliance on medical records. Data collection (density of the hematoma and specific types of the anticoagulants) and imaging reviews are less complete and accurate than a planned research. The assessment of neurological status requires detailed classification. In spite of these limitations, this study provides useful information to identify the minimally invasive surgery using the novel YL-1 puncture needle and summarize the risk factors of recurrence in CSDH, which can provide MIS and early therapeutic strategies for neurosurgeons.

## Figures and Tables

**Table 1:** Univariate analysis of recurrence factors of minimally invasive surgery in CSDH. Computer Tomography (CT); Magnetic Resonance Imaging (MRI).

	Recurrence (N = 33)	No Recurrence (N = 483)	*p*-Value
Gender (male–female)	30:3	399:84	0.218
Age (range)	68.48 ± 11.07	67.00 ± 11.79	0.482
History of head trauma (yes–no)	24:9	357:42	0.881
Past history:			
Anticoagulants (yes–no)	4:29	18:465	0.045
Disturbed liver function (yes–no)	1:32	10:573	0.520
History of heart disease (yes–no)	4:29	44:439	0.534
History of malignant tumors (yes–no)	2:31	20:463	0.644
History of diabetes (yes–no)	2:31	43:440	0.758
History of hemodialysis (yes–no)	0:33	6:477	0.614
History of coagulopathy (yes–no)	1:32	24:459	0.372
History of alcoholism (yes–no)	0:33	1:482	0.794
CT or MRI:			
Hematoma (unilateral–bilateral)	25:8	356:127	0.795
Mixed densities (yes–no)	15:18	250:233	0.511
Septum formation (yes–no)	0:33	16:467	0.615
Maximum thickness (mm)	21.59 ± 6.26	21.64 ± 7.47	0.960
Encephalatrophy (yes–no)	5:28	50:433	0.380
Postoperative treatments:			
Atorvastatin (yes–no)	25:8	335:148	0.504
Urokinase (yes–no)	29:4	433:50	0.836

**Table 2:** Logistic analysis of factors affecting recurrence in CSDH.

	B	Standard Error	Wals	df	*p*-Value	Exp (B)	95% CI
Anticoagulants	1.271	0.585	4.718	1	0.030	3.563	1.132–11.214

## Data Availability

These data can be requested from the corresponding author on reasonable request.

## Abbreviations

MIS	minimally invasive surgery
CSDH	chronic subdural hematoma
